# Associations between different body mass index and lung function impairment in Chinese people aged over 40 years: a multicenter cross-sectional study

**DOI:** 10.1186/s12890-024-02844-x

**Published:** 2024-01-11

**Authors:** Yumeng Tang, Lan Zhang, Shuzhen Zhu, Miaoyan Shen, Maowei Cheng, Fei Peng

**Affiliations:** Hubei Provincial Center for Disease Control and Prevention, Hubei, China

**Keywords:** Lung function impairment, Body mass index, Prevalence, Cross-sectional study

## Abstract

**Objective:**

The aim of this study was to explore the associations between different body mass index (BMI) levels and different lung function impairment (LFI) in Chinese people aged over 40 years.

**Methods:**

We used a multi-stage stratified cluster random sampling method to investigate 3000 residents aged over 40 years from 5 areas in Hubei province of China in 2019-20. The data on questionnaire, physical measurements, and spirometry of the participants were collected. The associations of different BMI levels with different LFI were analyzed using multivariate logistic regressions after complex weighting. The spirometry data were analyzed using one-factor analysis of variance (ANOVA), and post-hoc was performed using the least significance difference (LSD)-t test.

**Results:**

A total of 2860 subjects were included. The prevalence (95%*CI*) of obstructive lung disease (OLD), restrictive lung disease (RLD), mixed lung disease (MLD), chronic obstructive pulmonary disease (COPD), COPD mild, and COPD moderate/severe/very severe were 24.1% (95% CI: 22.2–26.2), 11.6% (95% CI: 10.3–12.9), 4.0% (95% CI: 3.3–4.8), 12.6% (95% CI: 11.0-14.1), 7.2% (95% CI: 6.0-8.4), and 5.3% (95% CI: 4.3–6.4) respectively. After multivariate adjustment, the risk of OLD, COPD, and COPD mild decreased with the increment of BMI levels (both *P* for trend < 0.05). When compared to the normal weight group, the overweight group and obese group were at lower risk of experiencing OLD than normal group, the ORs were 0.77 (95% CI: 0.59–0.99) and 0.59 (95% CI: 0.40–0.86) respectively. The obese group was at lower risk for people with COPD mild (OR: 0.42, 95%CI: 0.21–0.85). Participants in underweight group were more likely to experience COPD and COPD moderate/severe/very severe, the ORs were 2.82 (95% CI: 1.07–7.39) and 3.89 (95% CI: 1.28–11.87) respectively.

**Conclusion:**

Higher BMI levels were associated with an decreased risk of OLD and COPD. Obesity had a protective effect on lung function in OLD patients and COPD patients. However, there was no significant difference in RLD and MLD prevalence between different BMI groups.

**Supplementary Information:**

The online version contains supplementary material available at 10.1186/s12890-024-02844-x.

## Introduction

Lung function is a long-term predictor for overall survival rates and could be used as a tool in individual lung health assessment as well as general health assessment [1–3]. Lung function impairment (LFI) is a sign of early respiratory injury and a syndrome of progressive lung function decline. There are several lung diseases accompanied by LFI, such as obstructive lung disease (OLD), restrictive lung disease (RLD), mixed lung disease (MLD), chronic obstructive pulmonary disease (COPD), etc. It has been well documented that impaired lung function is associated with various adverse clinical outcomes such as all-cause mortality, stroke, heart failure, coronary heart disease, and cognitive impairment [1, 4–10]. The underlying causal mechanisms, such as chronic systemic inflammation, hypoxia, and right ventricular dysfunction have been considered to account for these associations [11–13].

Apart from the unremitting health problems for individuals, LFI has also become a major public health threat that deserves more attention. As the main and representative disease of LFI, COPD has caused significant morbidity and mortality with excessive health resource consumption and health expenditures, making it one of the major contributors for the rising burden of un-communicable chronic diseases in China [14, 15]. The prevalence and disease burden of COPD in China have received considerable attention. In a large, nationally representative sample of adults over 40 years, the prevalence of COPD was reported as high as 13.7% [16]. Meanwhile, the COPD prevalence at the provincial level in China have been published elsewhere [17–20]. However, the population-based studies on the prevalence of other lung diseases such as OLD, RLD, and MLD in China are very limited. Specifically, the studies focusing on the prevalence and spirometry parameters of different lung diseases by different subgroups have been lacking.

Many factors influence the lung function values such as age, sex, height, weight, ethnicity, etc., among which body mass index (BMI) plays an very important role. The relationship of BMI with lung function and COPD has been previously described in many studies, but the results were inconsistent. A number of studies revealed that low BMI and being underweight were linked to a reduced FEV1 and higher risk of COPD than normal body composition [21–23]. A positive association between BMI and lung function, measured using forced vital capacity (FVC) and forced expiratory volume in 1s (FEV1), has been demonstrated in a study of 22,743 participants [24]. However, the exact correlation between obesity and COPD remains controversial. There were studies indicating that overweight and obesity were protective factors for COPD [25–27], while a large cross-sectional population-based study considered them as risk factors for COPD [28]. Moreover, it has also been reported that there was no statistical significance between overweight or obesity and COPD [29–31]. Some findings suggested that poor exercise ability and muscle function in underweight people and oxidative stress as well as inflammation in obese people may be the factors contributing to the pathogenesis of LFI [23, 32, 33].

Despite the increasing number of studies exploring the associations between BMI and COPD, there is a lack of studies on the association between different BMI categories and lung function in patients with different lung diseases. There are also few epidemiological studies on the prevalence of OLD, RLD, MLD in China. In this study, we tried to provide an up-to-date estimate of the prevalence of COPD as well as other lung diseases (OLD, RLD, and MLD) in Hubei province of mid-China using data from a survey done in 2019-20, which was a part of a nationally cross-sectional study following a strict quality assurance and control programme to ensure data validity and reliability. Also, we aimed to examine the associations between different BMI categories and different LFIs as well as the effect of BMI on spirometric parameters.

## Methods

### Study population and design

This study was a part of nationwide cross-sectional study of China using the integrated national disease surveillance point (DSP) system from the Chinese Center for Disease Control and Prevention [16, 34, 35]. We conducted multi-center surveys using a complex, multistage, and probability sampling strategy from five DSPs in Hubei province of China from December 2019 to December 2020. Hubei province, located in the central part of China and the middle reaches of Yangtze River, has an area of 185,900 square kilometers and a population of nearly 60 million. In the first stage, three sub-districts/townships were randomly chosen in each DSP. Secondly, two neighboring communities/villages were randomly selected within each sub-districts/township. Thirdly, one group of villagers with more than 150 households was randomly chosen within each community/village. Finally, 100 households within each group of villagers were randomly selected. From each household, one family member aged over 40 years old was randomly selected using a Kish grid sampling approach [16]. Residents with cognitive/literal/mental disorders, pregnancy, cancers, or paraplegia were excluded. A representative sample of adults aged 40 years or older in Hubei Province in 2019-20 was enrolled. All participants provided written informed consent. The overall response rate defined according to the American Association for Public Opinion research was 99.8% in this study (Table S1) [36].

### Procedures

All participants were invited to an face-to-face interview by trained staff to obtain information on demographic characteristics, medical history, influencing factors of respiratory disease, and respiratory symptoms. Specifically, influencing factors of respiratory disease included smoking status and family history of lung diseases. Physical examination includes measurements of height, weight, waistline, blood pressure, and heart rate. Body weight was measured in light clothing to the nearest 0.1 kg with a calibrated balance beam scale. Height without shoes on was measured to the nearest 0.5 cm using a vertical ruler. Body mass index (BMI) was computed as the ratio of body weight (kg) to height squared (m^2^). Body weight status was defined as BMI based on World Health Organization guidelines. BMI status was classified as “low (< 18.5 kg/m^2^ ),” “normal (18.5–23.9 kg/m^2^),” “overweight (24.0–27.9 kg/m^2^ )” or “obese (≥ 28.0 kg/m^2^)” [37].

Spirometry was conducted based on the recommendations by the American Thoracic Society using calibrated spirometers (MasterScreen Pneumo, Jaeger, Germany) [16, 38]. Prebronchodilator and post-bronchodilator (15 min after administration of 400 µg salbutamol) forced expiratory volume in 1s (FEV1), and forced vital capacity (FVC) were measured. The detailed information on these procedures (including definition of other COPD-specific risk factors, full details of the spirometry method used, and a quality grade method for spirometry results) is described in the nationwide prevalence study [16]. Lung function parameters, including the forced vital capacity (FVC), the forced expiratory volume in the first second (FEV1), and the ratio of FEV1/FVC, were collected before and after using bronchodilator. The predicted values of FEV1 for normal lung function and the lower limited normal (LLN) of FEV1 from a nationwide study of reference values for spirometry in the Chinese population aged 4–80 years old [39]. The prediction equations include age, sex, height, and weight. All results were graded as A, B, C, D, and F based on acceptable operation and repeatability of FVC and FEV1. Grades of A, B, and C were regarded as acceptable for analyses. The severity of airflow limitation was measured by the absolute FEV1, the predicted FEV1, and by the ratio of absolute FEV1/the predicted percentage of FEV1 (FEV1%).

### Definitions

We defined different lung function impairments based on the collected FEV1 (L), FVC (L), the predicted FEV1 (%), the predicted FVC (%), FEV1/FVC ratio (%), and the predicted FEV1/FVC ratio (%). COPD was defined by a post-bronchodilator FEV1/FVC < 70% as per the recommendation of the 2018 Global Initiative for Chronic Obstructive Lung Disease (GOLD) [40]. The four GOLD severity grades are defned as GOLD stage I(mild, corresponding to FEV1 ≥ 80% predicted), GOLD stage II(moderate, FEV1 ≥ 50% to <80% predicted), GOLD stage III (severe, FEV1 ≥ 30% to <50% predicted), and GOLD stage IV (very severe disease, FEV1 < 30% predicted). For other respiratory impairments. According to the guidelines for pulmonary function tests (PFTs) published by Chinese Thoracic Society [41], obstructive lung disease (OLD), restrictive lung disease (RLD), and mixed lung disease (MLD) were defined. (1) OLD: FEV1/FVC < 92% of predicted value; FEV1 < 80% predicted, and FVC > 80% predicted; meet any one of the above two requirements. (2) RLD: FVC < 80% predicted. (3) MLD: simultaneously meeting the above (1)+(2) criteria.

### Statistical analysis

Statistical analyses were performed with SAS version 9.4 software (SAS Institute, Cary, NC). Categorical variables were expressed as number (n) or percentage (%) and continuous variables as mean ± standard deviation (SD). We used Rao-Scott χ^2^ test for categorical variables for comparison. We calculated p values for trend using Cochran-Armitage trend test for proportions. The association between BMI and different lung function impairments was evaluated using multivariable logistic regression after complex weighting, and odds ratios (ORs) for different lung function impairments were calculated after adjustment for age, residence, gender, education level, occupation, smoking status, family history of lung diseases. All hypothesis testing was judged at the 0.05 significance level (two-sided). The spirometry parameters (means and SD) were analyzed by univariate analysis of variance (ANOVA) using the Proc GLM procedures of SAS, and least-significant-difference (LSD) pairwise comparisons was used to analyze differences in spirometry parameters among different BMI categories.

## Results

### Characteristics of study subjects

**Fig. 1:**
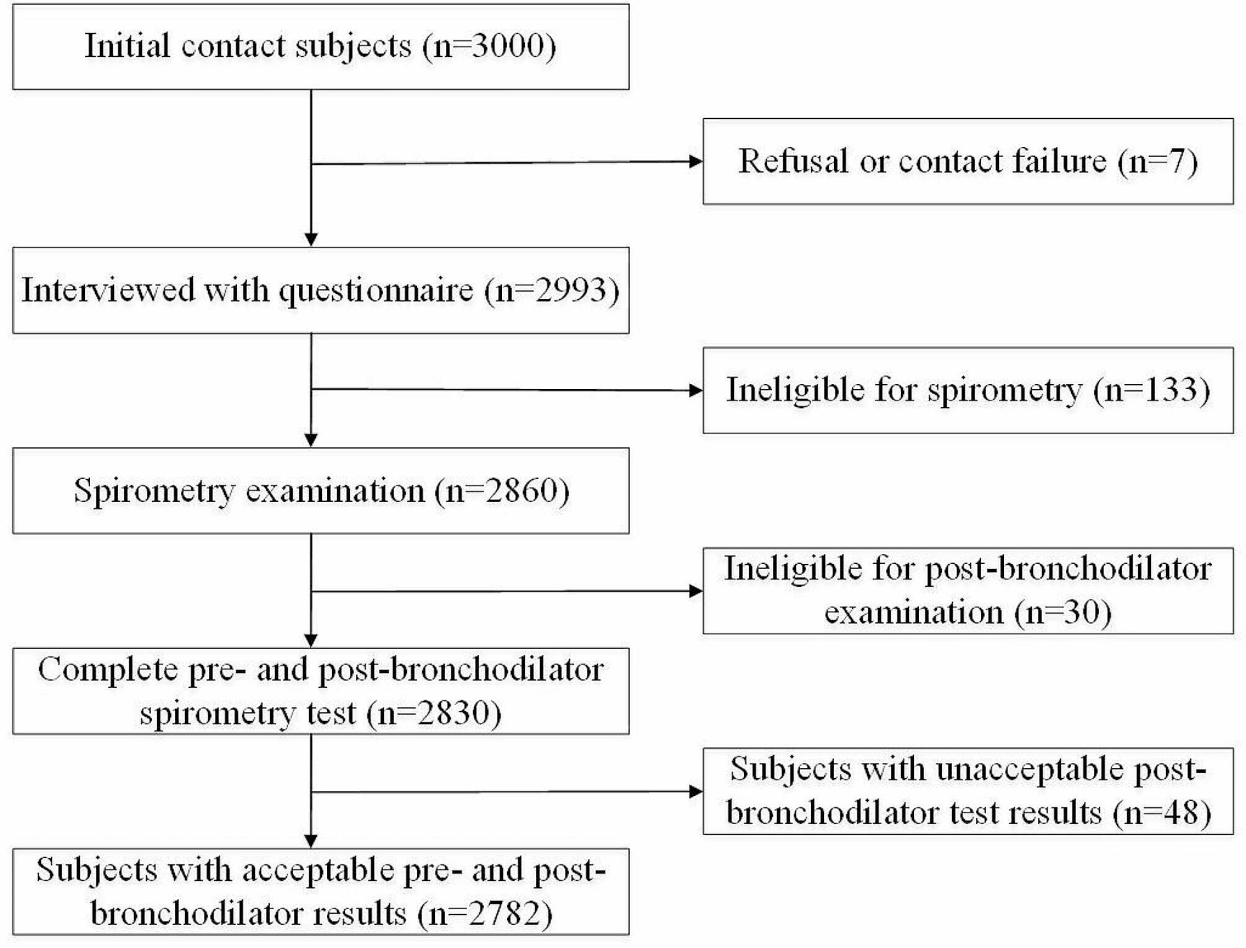
Flowchat on selection of participants in the study

Between December 2019 and December 2020, a total of 3000 subjects were contacted to take part in the survey, of whom 2993 (99.8%) were interviewed. 133 individuals were not eligible for spirometry. 2860 participants completed pre-bronchodilator spirometry examination, among which 2830 participants completed post-bronchodilator examinations. Subjects with unacceptable post-bronchodilator test results were excluded. In total, 2782 participants took part in the interview and had acceptable post-bronchodilator spirometry examinations (Fig. 1). Table 1 presents the demographic characteristics and exposures of 2860 study participants. The proportion of the age group of 50–59 years was the highest (42.8%). Participants from rural areas (68.1%) were more than those from urban areas (31.9%). For exposures of study participants, 1204 (39.9%) participants had smoking status, of whom 940 (30.9%) were current smokers, 264 (8.9%) were former smokers, 593 (22.8%) had family history of lung diseases.

**Table 1:** Demographic characteristics and exposures of study participants (n = 2860)

	Total†	OLD‡	RLD‡	MLD‡	COPD‡	COPD mild‡	COPD moderate/severe/very severe‡
Age group(years)							
40~	465 (17.6)	112 (22.4)	74 (9.6)	26 (3.2)	20 (4.3)	11 (1.7)	9 (2.6)
50~	1212 (42.8)	291 (21.8)	173 (9.4)	61 (2.8)	136 (10.4)	86 (5.7)	50 (4.7)
60~	851 (29.0)	240 (26.8)	169 (14.0)	64 (4.7)	141 (17.1)	87 (10.2)	54 (6.9)
≥ 70	332 (10.5)	99 (29.2)	98 (17.6)	45 (8.8)	75 (23.7)	53 (15.3)	22 (8.5)
p value for trend		0.025*	< 0.001***	< 0.001***	< 0.001***	< 0.001***	< 0.001***
Gender							
Male	1683 (56.9)	598 (33.8)	290 (11.3)	149 (5.7)	322 (19.1)	202 (11.1)	120 (8.0)
Female	1177 (43.1)	144 (11.4)	224 (12.0)	47 (1.8)	50 (4.0)	35 (2.2)	15 (1.8)
p value		< 0.001***	0.627	< 0.001***	< 0.001***	< 0.001***	< 0.001***
Residence							
Urban	860 (31.9)	171 (14.9)	153 (8.0)	46 (1.6)	70 (5.9)	47 (3.9)	23 (2.0)
Rural	2000 (68.1)	571 (28.5)	361 (13.3)	150 (5.2)	302 (15.7)	190 (8.8)	112 (6.9)
p value		< 0.001***	< 0.001***	< 0.001***	< 0.001***	< 0.001***	< 0.001***
Education level							
Primary and lower	1624 (53.3)	401 (22.7)	326 (12.8)	126 (4.7)	215 (13.7)	142 (8.5)	73 (5.2)
Secondary school	920 (33.4)	257 (26.6)	135 (10.4)	49 (3.2)	119 (12.4)	69 (6.3)	50 (6.1)
Higher or further education	316 (13.3)	84 (23.9)	53 (9.7)	21 (3.3)	38 (8.8)	26 (4.7)	12 (4.0)
p value for trend		0.334	0.055	0.101	0.044*	0.015*	0.806
Occupation							
Agriculture	1810 (60.0)	485 (26.1)	277 (12.3)	106 (4.4)	255 (14.7)	150 (8.0)	105 (6.7)
Non-agriculture	590 (22.0)	166 (25.7)	155 (10.6)	61 (3.7)	81 (11.5)	65 (8.5)	16 (3.0)
Unemployed and houseworker	409 (15.8)	73 (13.5)	67 (9.4)	23 (3.0)	29 (6.1)	17 (2.2)	12 (3.9)
Retired	51 (2.2)	18 (29.7)	15 (18.9)	6 (4.7)	7 (13.2)	5 (9.9)	2 (3.4)
p value		< 0.001***	0.193	0.515	0.004**	0.003**	0.053
Smoking status#							
Never smoker	1654 (60.2)	275 (13.8)	314 (11.7)	85 (2.5)	102 (5.1)	67 (2.8)	35 (2.2)
Former smoker	264 (8.9)	101 (38.7)	57 (15.1)	33 (9.0)	68 (27.7)	43 (15.7)	25 (12.0)
Current smoker	940 (30.9)	366 (40.0)	143 (10.4)	78 (5.6)	202 (23.0)	127 (13.5)	75 (9.5)
p value		< 0.001***	0.154	< 0.001***	< 0.001***	< 0.001***	< 0.001***
Family history of lung diseases							
No	2267(77.2)	551(22.7)	444 (12.4)	166 (4.2)	270 (11.6)	181 (6.7)	89 (4.8)
Yes	593(22.8)	191(29.0)	70 (9.1)	30 (3.5)	102 (16.0)	56 (8.9)	46 (7.1)
p value		0.012*	0.054	0.451	0.019*	0.137	0.078
Total	2860 (100.0)	742 (24.1)	514 (11.6)	196 (4.0)	372 (12.6)	237 (7.2)	135 (5.3)

*Note*: †Data are presented as n (%). ‡Data are presented as n (prevalence rate). #Data missing for smoking status (n = 2). *P<0.05; **P<0.01; ***P<0.001.

*Abbreviations*: OLD, obstructive lung disease; RLD, restrictive lung disease; MLD, mixed lung disease; COPD, chronic obstructive pulmonary disease

### Prevalence of COPD and other lung function impairment

Among 2860 participants who had pre-bronchodilator results, the prevalence of OLD, RLD, and MLD were 24.1% (95% CI: 22.2–26.2), 11.6% (95% CI: 10.3–12.9), and 4.0% (95% CI: 3.3–4.8) respectively. Among 2830 participants who had both pre-bronchodilator and post-bronchodilator results, the prevalence of COPD, COPD mild, and COPD moderate/severe/very severe was 12.6% (95% CI: 11.0-14.1), 7.2% (95% CI: 6.0-8.4), and 5.3% (95% CI: 4.3–6.4) respectively. The age-specific prevalence of OLD, RLD, MLD, COPD, COPD mild, and COPD moderate/severe/very severe rose significantly with increasing age (*P* < 0.05) and were significantly different between urban and rural residence (*P* < 0.001). Univariate analysis demonstrated that lower education level (primary school or lower) was correlated significantly with higher COPD and COPD mild prevalence in this population. The prevalence of those above differed significantly between men and women (*P* < 0.001) except for RLD (*P* > 0.05). Regarding smoking status, the prevalence of OLD, MLD, COPD, COPD mild, and COPD moderate/severe/very severe were significantly higher in current smokers and former smokers than never smokers, while the prevalence of RLD was not significantly different. Subjects with family history of lung diseases had significantly higher prevalence in OLD and COPD than those without (*P* < 0.05).

### Associations between different BMI and lung function impairment

When BMI was used as a qualitative variable, the trend analysis showed that lower BMI was positively correlated with higher prevalence of OLD, COPD, and COPD mild (*P* < 0.05). For people diagnosed with COPD moderate/severe/very severe, the prevalence was the highest in underweight group (16.1%) compared to other BMI groups, but the p value for trend was not significant (Table 2). However, there was no significant difference in RLD and MLD prevalence between different BMI groups (*P* > 0.05). Table 3 displayed associations of different BMI categories with lung function impairment. When the normal weight group was designated as the reference level after using adjusted logistic analysis, the overweight group and obese group were at lower risk of experiencing OLD than normal group, the ORs were 0.77 (95% CI: 0.59–0.99) and 0.59 (95% CI: 0.40–0.86) respectively. However, the overweight group was at higher risk of experiencing RLD (OR: 1.40, 95%CI: 1.04–1.87). Compared to normal group, participants in underweight group were more likely to experience COPD and COPD moderate/severe/very severe, the ORs were 2.82 (95% CI: 1.07–7.39) and 3.89 (95% CI: 1.28–11.87) respectively. The obese group was at lower risk for people with COPD mild (OR: 0.42, 95%CI: 0.21–0.85). The negative dose response relationships between different BMI groups and the risk of OLD, COPD, and COPD mild were statistically significant (*P* value for trend < 0.05).

**Table 2:** Distribution of respiratory impairment among subjects with different body mass index

	OLD	RLD	MLD	COPD	COPD mild	COPD moderate/severe/very severe
Underweight	13 (33.4)	9 (7.7)	3 (1.6)	9 (25.8)	4 (9.7)	5 (16.1)
Normal	381 (27.2)	240 (10.9)	102 (4.4)	215 (14.9)	148 (9.4)	67 (5.5)
Overweight	272 (21.4)	200 (12.6)	74 (4.0)	119 (10.1)	68 (5.4)	51 (4.7)
Obese	76 (19.4)	65 (12.1)	17 (3.0)	29 (8.9)	17 (4.1)	12 (4.8)
p value for trend	0.002**	0.239	0.39	0.001**	< 0.001***	0.24

*Note*: Data are presented as n (prevalence rate). *P<0.05; **P< 0.01; ***P<0.001.

*Abbreviations*: OLD, obstructive lung disease; RLD, restrictive lung disease; MLD, mixed lung disease; COPD, chronic obstructive pulmonary disease

**Table 3:** Adjusted prevalence ratios (95% confidence interval) for different categories of respiratory impairment by body mass index

	Underweight	Normal	Overweight	Obese	p value for trend
OLD	1.94 (0.77~4.90)	1.00	**0.77 (0.59~0.99)**	**0.59 (0.40~0.86)**	< 0.001***
RLD	0.79 (0.29~2.14)	1.00	**1.40 (1.04~1.87)**	1.25 (0.81~1.93)	0.059
MLD	0.49 (0.13~1.91)	1.00	1.10 (0.69~1.74)	0.75 (0.36~1.56)	0.807
COPD	**2.82 (1.07~7.39)**	1.00	0.76 (0.55~1.06)	0.59 (0.34~1.02)	0.006**
COPD mild	1.36 (0.30~6.19)	1.00	0.69 (0.46~1.01)	**0.42 (0.21~0.85)**	0.004**
COPD moderate/severe/very severe	**3.89 (1.28~11.87)**	1.00	0.95 (0.58~1.57)	0.94 (0.44~2.02)	0.397

*Note*: Adjusted for age, residence, gender, education level, occupation, smoking status, family history of lung diseases. *P<0.05; **P<0.01; ***P<0.001.

*Abbreviations*: OLD, obstructive lung disease; RLD, restrictive lung disease; MLD, mixed lung disease; COPD, chronic obstructive pulmonary disease

### The effect of BMI on lung function in lung function impairments

To investigate the effect of BMI on lung function of different lung function impairment categories, we compared the spirometry parameters using ANOVA analysis. As shown in Table 4, there were statistically significant differences of lung function measurements for different BMI categories in people with OLD, COPD, COPD mild, and COPD moderate/severe/very severe. For people with OLD, the mean value of FEV1/FVC ratio and FEV1/FVC%predicted increased significantly from underweight to obese group (*P <* 0.05). For people with RLD, the mean value of FEV1/FVC ratio and FEV1/FVC%predicted in overweight and obese group were significantly higher when compared to the normal group (*P <* 0.05). For people with MLD, there was no significant difference in spirometry parameters between different BMI categories (*P >* 0.05). For people with COPD, significant differences were observed in the mean value of FEV1, FEV1%predicted, FEV1/FVC ratio and FEV1/FVC%predicted between different BMI groups. Specifically, the FEV1 and FEV1%predicted in underweight group were significantly lower than normal group, while the FEV1 in underweight group was also significantly lower than overweight group and obese group (*P* < 0.05). For people with COPD moderate/severe/very severe, the FEV1/FVC ratio and FEV1/FVC%predicted in overweight group and obese group were significantly higher than those in normal group (*P* < 0.05).

**Table 4:** Spirometry parameters (means and SD) for different categories of respiratory impairment

	FEV1, L	FEV1, %predicted	FVC, L	FVC, %predicted	FEV1/FVC ratio	FEV1/FVC ratio, %predicted
OLD						
Underweight	2.05 ± 0.64	80.75 ± 24.22	3.04 ± 0.79	92.88 ± 18.72	59.20 ± 11.65	75.11 ± 14.31
Normal	2.59 ± 0.84†	92.28 ± 26.06	3.27 ± 0.98	92.59 ± 24.73	65.30 ± 9.03†	81.99 ± 11.03
Overweight	2.61 ± 0.77†	90.76 ± 23.27	3.35 ± 1.00	92.60 ± 22.96	66.30 ± 8.11†#	82.99 ± 9.82†
Obese	2.59 ± 0.72†	91.17 ± 23.42	3.32 ± 0.93	92.61 ± 24.50	67.64 ± 8.02†#	84.43 ± 9.91†
p value	0.227	0.235	0.95	0.919	< 0.001***	< 0.001***
RLD						
Underweight	2.46 ± 0.70	91.91 ± 24.20	2.27 ± 0.49	66.24 ± 8.35	74.45 ± 13.41	93.55 ± 16.70
Normal	2.43 ± 0.83	92.06 ± 24.33	2.20 ± 0.60	65.79 ± 12.53	71.64 ± 11.92	90.50 ± 14.99
Overweight	2.34 ± 0.76	89.81 ± 22.98	2.22 ± 0.55	67.37 ± 10.74	73.30 ± 11.05#	92.37 ± 13.82#
Obese	2.37 ± 0.72	90.06 ± 22.30	2.25 ± 0.48	68.10 ± 10.50	75.74 ± 9.78#	95.14 ± 12.16#
p value	0.086	0.731	0.57	0.205	0.12	0.142
MLD						
Underweight	2.42 ± 0.86	94.29 ± 42.21	2.22 ± 0.44	65.94 ± 7.64	59.86 ± 3.69	74.77 ± 5.90
Normal	2.45 ± 0.99	88.49 ± 30.83	2.13 ± 0.69	60.39 ± 15.39	61.56 ± 11.39	77.71 ± 14.17
Overweight	2.37 ± 0.90	86.52 ± 29.76	2.20 ± 0.59	62.92 ± 12.84	62.05 ± 9.48	78.30 ± 11.80
Obese	2.57 ± 0.94	95.48 ± 32.17	2.24 ± 0.56	66.00 ± 12.87	64.25 ± 10.95	80.48 ± 13.76
p value	0.474	0.263	0.848	0.226	0.784	0.804
COPD						
Underweight	1.79 ± 0.52	69.00 ± 17.20	2.89 ± 0.90	87.16 ± 23.85	58.16 ± 15.52	73.97 ± 19.27
Normal	2.48 ± 0.84†	90.59 ± 28.34†	3.22 ± 0.91	92.59 ± 23.25	64.46 ± 10.75	81.45 ± 13.61
Overweight	2.24 ± 0.69†#	80.95 ± 23.92#	3.23 ± 0.92	91.36 ± 21.51	63.04 ± 9.72	79.46 ± 12.28
Obese	2.52 ± 0.91†	87.47 ± 29.39	3.34 ± 0.88	92.09 ± 19.96	65.10 ± 11.21	81.66 ± 14.24
p value	< 0.001***	< 0.001***	0.849	0.881	0.004**	0.006**
COPD mild						
Underweight	2.16 ± 0.26	84.33 ± 5.82	2.80 ± 1.25	82.89 ± 33.60	68.55 ± 13.99	87.73 ± 16.82
Normal	2.86 ± 0.68†	104.59 ± 20.95	3.39 ± 0.88	97.67 ± 22.70	68.91 ± 7.35	87.13 ± 9.26
Overweight	2.61 ± 0.53	95.98 ± 16.37#	3.40 ± 0.93	98.01 ± 21.83	67.41 ± 6.21	85.08 ± 7.86
Obese	3.02 ± 0.76†	104.82 ± 23.28	3.61 ± 0.80†	99.48 ± 17.16	69.19 ± 7.73	86.65 ± 10.23
p value	0.007**	0.014*	0.61	0.498	0.378	0.417
COPD moderate/severe/very severe						
Underweight	1.49 ± 0.49	56.74 ± 11.97	2.97 ± 0.65	90.58 ± 16.06	49.85 ± 11.86	62.95 ± 13.77
Normal	1.65 ± 0.50	59.66 ± 14.70	2.85 ± 0.86	81.35 ± 20.47	54.63 ± 10.57	68.88 ± 13.30
Overweight	1.75 ± 0.57	60.92 ± 16.57	3.00 ± 0.85	82.49 ± 17.67	57.22 ± 10.52#	71.97 ± 13.15#
Obese	1.81 ± 0.60	62.89 ± 16.91	2.96 ± 0.88	81.63 ± 19.56	59.32 ± 13.06†#	74.58 ± 16.45#
p value	0.812	0.735	0.768	0.605	0.045*	0.029*

*Note*: †*P* < 0.05 indicating the statistical difference comparing with underweight group. #*P* < 0.05 indicating the statistical difference comparing with normal group. *P<0.05; **P<0.01; ***P<0.001.

*Abbreviations*: OLD, obstructive lung disease; RLD, restrictive lung disease; MLD, mixed lung disease; COPD, chronic obstructive pulmonary disease; FEV1, forced expiratory volume in the first second; FVC, forced vital capacity; %predicted, percentage of predicted

## Discussion

This population-based and spirometry-based study followed a stringent quality-control method which is the first survey of COPD and other lung diseases in Hubei province of mid-China. As a part of the nationwide survey, the study allows us to make a comparison between COPD prevalence at provincial level and national estimation in China. As a province located in the central part of China, the prevalence of COPD of Hubei province (12.6%) in our study was higher than the prevalence of central China (10.2%) reported in the national survey, but was lower than the national prevalence (13.6%) [16]. Compared to other regions of China, the prevalence was lower than Kashi region, northwestern China (17.01%), but was higher than Anhui province (9.8%), Hainan province (5.07%), Jiangsu province (11.8%), and Shenzhen municipality (5.92%) among adults aged over 40 years [17, 19, 20, 42, 43]. A Global Burden of Disease Study of COPD in Hubei province showed that although the mortality of COPD in Hubei has been reduced, the absolute number of COPD cases is increasing [44]. Therefore, sustained attention to COPD control and ongoing surveillance are of great importance. As for other lung diseases, very little is currently known about the prevalence of OLD, RLD, and MLD in China as well as other countries. To the best of our knowledge, this is the first to report the prevalence of OLD (24.1%), RLD (11.6%), and MLD (4.0%) among the sampled people in China. A population-based Korea National Health and Nutrition Examination Survey (KNHANES) reported that the RLD was detected in 11.3% and OLD was detected in 13.2% among 16,151 subjects aged over 40 years [45, 46]. A national spirometric surveillance data in the United States showed that 13.5% of participants had OLD and 6.5% had RLD [47]. Both OLD, RLD, and COPD impose a significant burden on individuals through progressive symptoms, acute pulmonary exacerbations, worsening quality of life, and premature death [48].

The higher smoking exposure in men than women may explain the reason why the prevalence of OLD, MLD, and COPD were significantly higher in men than women [49]. It is well known that smoking habits are important determinants in reduced lung function and developed COPD [50]. The higher prevalence rates of OLD, RLD, MLD, and COPD in rural areas than those in urban areas can also be explained by the fact that cigarette smoking is more prevalent in rural areas [51]. Therefore, smoking may be the primary cause behind the observed disparities between different regions and different gender. Nevertheless, there was no significant disparity in RLD prevalence between male and female as well as with different smoking status. A community-based, cross-sectional study conducted in Taiwan showed that smoking was independently associated with obstructive or mixed types of lung impairment, but not for restrictive type [52]. Another longitudinal study revealed that individuals with the obstructive-only and mixed patterns, but not the restrictive-only pattern, had a significanly higher prevalence of adult smoking compared with the reference pattern. A Italian mulicentric study also proved that smoking was associated with obstructive ventilatory pattern, but not with restrictive ventilatory capacity [53]. It was reported that smoking would harm the airways with less than 2 mm internal diameter, so it results in airway obstruction [54]. The restrictive-only pattern might characterize individuals with poor lung development in childhood, but was found to have true lung restriction by middle age [55].

The effect of body mass index on the lung function have received considerable attention. It is widely demonstrated that being underweight or losing weight increased the risk of COPD exacerbation which leads to reduced lung function and increased risk of mortality [21–24, 27]. In our study, we found that underweight patients had higher prevalence of OLD and COPD than other BMI groups. Being underweight was shown to be a risk factor for COPD as well as COPD moderate/severe/very severe. This result is consistent with that of a cross-sectional study of 13,023 participants in low- and middle-income countries, which showed that individuals with lower BMI were more likely to have COPD and had lower lung function compared to those in higher BMI [21]. Although the exact cause of unplanned weight loss with a reduced BMI in COPD has not been clearly understood, the underweight patients might have malnutrition, low muscularity and weak resistance against respiratory infection [56]. Also, Rawal and Yadav (2015) concluded that several factors such as a raised resting energy expenditure, inflammation, hypoxia and medication use may contribute to the increasing risk of COPD [57]. On the other hand, the causal relationship between underweight and COPD can also work in the opposite direction. COPD can lead to weight loss and poor nutritional status over a prolonged period of time through appetite loss, diminished general physical activity, a depressive tendency, dyspnea while eating, enhanced energy expenditure due to increased work of breathing, and increased production of inflammatory cytokines [58]. A recent study has estimated that 25–40% of COPD patients are underweight while 35% of patients have severely low fat-free mass index [57]. Therefore, low body weight have been recognized as unfavorable prognostic factors in patients with COPD, especially in those with COPD moderate/severe/very severe level.

Another finding in our study was that the negative dose-response relationship between BMI levels and risk of OLD was significant among participants. Compared to participants with normal BMI, those who were overweight and obese have decreased risk for OLD. Also, participants with obesity had decreased risk for COPD mild. A meta-analysis of thirty articles with 1,578,449 participants concluded that overweight and obesity might reduce the risk of COPD while underweight might increase the risk of COPD [59]. A cross-sectional study of 32,033 subjects also showed that slight obesity was a protective factor for lung function in people at risk of COPD, but being severely obese were associated with reduced lung function [60]. The reduced risk of OLD and COPD in overweight or obese participants might be explained by several mechanisms. Firstly, patients with OLD or COPD need high respiratory muscle mass to deal with increased airway. resistance and airflow obstruction [61]. Secondly, obese COPD patients has the protective effect of higher fat-free mass (FFM), which is a substitute for skeletal muscle mass [62]. Moreover, obese COPD patients have smaller expiratory reserve volume and end-expiratory lung volume [63]. Although being overweight or obesity seemed to have protective effect on the prevalence of OLD and COPD, the mechanisms underlying the “obesity paradox” require further studies and elucidation. On the other hand, being overweight increased the risk of RLD. These findings in our study can be confirmed by a longitudinal study, which showed that adult obesity was significantly more prevalence in individuals with the restrictive-only pattern and less prevalent in the obstructive-only pattern, but was not associated with the mixed pattern [55]. Another longitudinal population-based study from Vienna also revealed that restrictive lung function was strongly modified by high fat mass, central obesity, and adiposity [64]. It has been implied that BMI increase may mostly relate to the development of a restrictive rather than obstructive lung function pattern [65].

We conducted further analysis about spirometry parameters to explore the effect of BMI on lung function for different lung diseases. Spirometry is considered as a tool in various clinical scenarios, including the diagnosis of lung diseases, the monitoring of disease progression, and the assessment of its severity. The data obtained via spirometry can be used in order to detect abnormalities in individuals, among which FVC, FEV1, and the ratio of FEV1 to FVC are commonly used to assess lung functions. In our study, FEV1 and FVC of patients with OLD and COPD from underweight groups were the lowest compared to other BMI groups. The same finding was found in two studies that low BMI was associated with a lower FEV1 and FVC [66, 67]. Our study extended the findings to show that low BMI is associated with decreased lung function represented by parameters such as FEV1, predicted FEV1(%), FVC, and predicted FVC(%) in people with OLD and COPD, but not in people with RLD and MLD. The association between overweight/obesity and spirometry parameters remains controversial. A positive and negative association between overweight/obesity and FEV1/FVC have been both reported before [21, 24, 32, 60, 63]. In our study, significant higher FEV1/FVC was found in overweight/obesity than normal BMI group in people with OLD and COPD moderate/severe/very severe. For people with RLD, the FEV1/FVC and FEV1/FVC%predicted of overweight and obesity group were significantly higher than those of normal BMI group. Study by Jing Zhu also found that increased BMI had a protective effect on lung function in COPD GOLD 3–4 grade, which was consistent with our result [68]. Since nutritional status plays a significant role in the lung function in later stage COPD patients, the prognostic value of BMI was particularly convincing in patients with severe COPD [69].

Our study does have a major strength in our ability to use spirometry measurements for identifying different lung function impairment. Of note, there were some limitations to our study. First, it was a cross-sectional study, so we cannot evaluate any temporal relationships and establish any causality. Second, the different reference equations and diagnostic criteria may lead to overdiagnosis or underdiagnosis of lung function impairment. Third, although the sampled size was not small, the relatively smaller sample size in several subgroups could limit the estimated precision. Moreover, we cannot eliminate the possibility that we misclassified people with different lung diseases.

## Conclusion

Our results in this study indicated that lower BMI was positively correlated with higher prevalence of OLD and COPD. Obesity had a protective effect on lung function in OLD patients and COPD patients. However, there was no significant difference in RLD and MLD prevalence between different BMI groups. Future longitudinal studies are needed to clarify the direction of the association between BMI and lung function.

### Electronic supplementary material

Below is the link to the electronic supplementary material.

**Supplementary Material 1:** **Table S1**. American Association for Public Opinion Research outcome rate calculator (Panel of in-person household surveys)

## Data Availability

The data analyzed in the current study are not publicly available but may be made available from the corresponding authors on reasonable request.
