# Supplementary material for: Associations between different body mass index and lung function impairment in Chinese people aged over 40 years: a multicenter cross-sectional study
Source: BMC Pulm Med. 2024 Jan 11;24:30. doi: 10.1186/s12890-024-02844-x (PMC10785338; doi:10.1186/s12890-024-02844-x)
Supplement: Supplementary file 1 — Supplementary Material 1: Table S1. American Association for Public Opinion Research outcome rate calculator (Panel of in-person household surveys) [file 12890_2024_2844_MOESM1_ESM.docx]

**Table S1.** American Association for Public Opinion Research outcome rate calculator (Panel of in-person household surveys).

|  | Final Disposition Codes^†^ | COPD survey^†^ |
| --- | --- | --- |
| Interview (Category 1) | 1.0 |  |
| Complete | 1.1 | 2860 |
| Partial | 1.2 | 133 |
| Eligible, non-interview (Category 2) | 2.0 |  |
| Refusal and breakoffs | 2.10 | 7 |
| Non-contact | 2.20 | 0 |
| Other, non-refusals | 2.30 | 0 |
| Total sample used |  | 3000 |
| I=Complete Interviews (1.1) |  | 2860 |
| P=Partial Interviews (1.2) |  | 133 |
| R=Refusal and break off (2.10) |  | 7 |
| NC=Non-contact (2.20) |  | 0 |
| O=Other (2.30) |  | 0 |
| Response Rate 1 |  |  |
| I/((I+P) + (R+NC+O) + (UH+UO)) |  | 0.947 |
| Response Rate 2 |  |  |
| (I+P)/((I+P) + (R+NC+O) + (UH+UO)) |  | 0.999 |
| Response Rate 3 |  |  |
| I/((I+P) + (R+NC+O) + e(UH+UO)) |  | 0.947 |
| Response Rate 4 |  |  |
| (I+P)/((I+P) + (R+NC+O) + e(UH+UO)) |  | 0.999 |
| Cooperation Rate 1 |  |  |
| I/((I+P)+R+O)) |  | 0.947 |
| Cooperation Rate 2 |  |  |
| (I+P)/((I+P)+R+O)) |  | 0.999 |
| Cooperation Rate 3 |  |  |
| I/((I+P)+R)) |  | 0.947 |
| Cooperation Rate 4 |  |  |
| (I+P)/((I+P)+R)) |  | 0.999 |
| Refusal Rate 1 |  |  |
| R/((I+P)+(R+NC+O) + UH + UO)) |  | 0.001 |
| Refusal Rate 2 |  |  |
| R/((I+P)+(R+NC+O) + e(UH + UO)) |  | 0.001 |
| Refusal Rate 3 |  |  |
| R/((I+P)+(R+NC+O)) |  | 0.001 |
| Contact Rate 1 |  |  |
| ((I+P)+R+O)/ ((I+P)+R+O+NC+ (UH + UO)) |  | 1.000 |
| Contact Rate 2 |  |  |
| ((I+P)+R+O) / ((I+P)+R+O+NC + e(UH+UO)) |  | 1.000 |
| Contact Rate 3 |  |  |
| ((I+P)+R+O) / ((I+P)+R+O+NC) |  | 1.000 |

^†^ Contents listed in the original table but not applicable for this survey were not listed.
